# Transcriptome-Wide Analysis of *Botrytis elliptica* Responsive microRNAs and Their Targets in *Lilium Regale* Wilson by High-Throughput Sequencing and Degradome Analysis

**DOI:** 10.3389/fpls.2017.00753

**Published:** 2017-05-18

**Authors:** Xue Gao, Qi Cui, Qin-Zheng Cao, Qiang Liu, Heng-Bin He, Dong-Mei Zhang, Gui-Xia Jia

**Affiliations:** ^1^Beijing Key Laboratory of Ornamental Plants Germplasm Innovation and Molecular Breeding, National Engineering Research Center for Floriculture, Beijing Laboratory of Urban and Rural Ecological Environment and College of Landscape Architecture, Beijing Forestry UniversityBeijing, China; ^2^Shanghai Academy of Landscape Architecture Science and PlanningShanghai, China; ^3^Shanghai Engineering Research Center of Landscaping on Challenging Urban SitesShanghai, China

**Keywords:** *lily* (*Lilium regale* Wilson), *Botrytis elliptica*, miRNA, high-throughput sequencing, degradome

## Abstract

MicroRNAs, as master regulators of gene expression, have been widely identified and play crucial roles in plant-pathogen interactions. A fatal pathogen, *Botrytis elliptica*, causes the serious folia disease of lily, which reduces production because of the high susceptibility of most cultivated species. However, the miRNAs related to *Botrytis* infection of lily, and the miRNA-mediated gene regulatory networks providing resistance to *B. elliptica* in lily remain largely unexplored. To systematically dissect *B. elliptica*-responsive miRNAs and their target genes, three small RNA libraries were constructed from the leaves of *Lilium regale*, a promising Chinese wild *Lilium* species, which had been subjected to mock *B. elliptica* treatment or *B. elliptica* infection for 6 and 24 h. By high-throughput sequencing, 71 known miRNAs belonging to 47 conserved families and 24 novel miRNA were identified, of which 18 miRNAs were downreguleted and 13 were upregulated in response to *B. elliptica*. Moreover, based on the lily mRNA transcriptome, 22 targets for 9 known and 1 novel miRNAs were identified by the degradome sequencing approach. Most target genes for *elliptica*-responsive miRNAs were involved in metabolic processes, few encoding different transcription factors, including *ELONGATION FACTOR 1 ALPHA* (*EF1a*) and *TEOSINTE BRANCHED1/CYCLOIDEA/PROLIFERATING CELL FACTOR 2* (*TCP2*). Furthermore, the expression patterns of a set of *elliptica*-responsive miRNAs and their targets were validated by quantitative real-time PCR. This study represents the first transcriptome-based analysis of miRNAs responsive to *B. elliptica* and their targets in lily. The results reveal the possible regulatory roles of miRNAs and their targets in *B. elliptica* interaction, which will extend our understanding of the mechanisms of this disease in lily.

## Introduction

Lily (*Lilium* spp.) is one of the most valuable commercial market flower bulbs in the world, mainly owing to its various ornamental functions as a cut flower or a potted plant. However, the production and reproduction of lily have been impeded by *Botrytis* species, an aggressive fungal pathogen. It can infect more than 200 plant species, of which *Botrytis elliptica* specializes in lily (Van Baarlen et al., 2007; Weiberg et al., 2013). This pathogen infects leaves, stems and flowers of lily during plant growth as well as survival. To limit yield losses caused by gray mold, agronomic, genetic, and biological approaches have been proposed, however, cultivars are still threatened (Angelini et al., 2014). Although certain cultivars show some level of resistance to *B. elliptica*, few *Lilium* species or lily cultivars are absolutely immune to *B. elliptica* infection (Kohl et al., 1995; Lu et al., 2007). *L. regale* Wilson is native to western Sichuan Province in China, which is specifically distributed in the river basin of Minjiang. It was found to possess extremely high resistance to viruses and fungal pathogens, such as *F. oxysporum* and *B. elliptica* (Lim et al., 2003; Du et al., 2014). Therefore, it has been widely regarded as a main plant to investigate the disease-resistant mechanism of lily. For example, Rao et al. (2013) selected *L. regale* as materials to isolate the genes differentially expressed in a resistant to *F. oxysporum* reaction using the suppression subtractive hybridization. Liu et al. (2016) fully investigated the reference genes of *L. regale* under different abiotic and biotic stresses because of the advantages in breeding for further research. Thus, *L. regale* was utilized as experimental material in our study to explore the biochemical and genetic bases of resistance to *B. elliptica*.

MicroRNAs (miRNAs), ~21–24 nucleotides (nt) in length, are a subset of small, endogenous non-coding RNAs that mediate gene expression at the post-transcriptional level by targeting mRNAs for degradation or translational repression (Carrington and Ambros, 2003; Jover-Gil et al., 2005). Increasing evidences indicates that miRNAs are intimately involved in developmental processes (Wu et al., 2009; Kang et al., 2012; Zhang et al., 2012), flowering (Wang et al., 2012, 2014; Luo et al., 2013), and also many adaptive responses to both abiotic and biotic stresses in plants (Jones-Rhoades et al., 2006; Zhao C. et al., 2015; Niu et al., 2016; Shu et al., 2016). Roles of miRNAs in plant defense responses have been identified in *Solanum lycopersicum* (Jin et al., 2012; Jin and Wu, 2015), *Arabidopsis thaliana* (Kurubanjerdjit et al., 2014), *Paulownia fortune* (Niu et al., 2016), *Arachis hypogaea* L. (Zhao C. et al., 2015), and *Carica papaya* (Abreu et al., 2014), showing that some miRNAs can silence genes involved in immunity. For example, by using microarray technology, three known miRNAs (miR160, miR169, and miR171) were identified to exhibit differential expression profiles under *Botrytis cinerea* infection, which regulated adaptions of tomato seedlings at the post-transcriptional level (Jin et al., 2012). Recently, high-throughput sequencing technologies have provided unprecedented opportunities to generate both known and novel miRNAs by constructing small RNA libraries. Thus, it has been extensively applied to discovering miRNA in plant disease. For instance, via high-throughput sequencing, 41 significantly differentially expressed known miRNAs and 39 novel miRNAs were identified in response to *Rhizoctonia solani* (Luo et al., 2015). Among these miRNAs, miR171, miR408, and miR398 were found to be upregulated at a high level after *R. solani* treated 6, 12, and 24 h (Luo et al., 2015). Besides, Luan et al. (2015) found a total 70 miRNAs which were manifested to change significantly in samples treated with *P. infestans* using high-throughput sequencing, including 50 downregulated miRNAs and 20 upregulated miRNAs. Moreover, miRNAs that may be involved in plant disease can be further studied combined with the analysis of target genes. In peanut, the accumulation of some miRNAs was altered after infection with bacterial wilt disease, and more than 10% of their targets were shown to be defense response genes (Zhao C. et al., 2015). Functional identification and analysis of miRNAs and their targets in response to pathogen infection could provide valuable information for understanding the mechanisms underlying disease resistance. Concerning gray mold disease, *Solanum lycopersicum* (Jin and Wu, 2015) and *Paeonia lactiflora* (Zhao D. et al., 2015) have been reported to have miRNAs with functional roles in resistance to *B. cinerea*. However, the diversity of miRNAs and their roles in response to *B. elliptica*-infection in lily remain largely unexplored.

Given their huge and complex genomes, research on lily miRNA has been delayed relative to that on model plants, such as *Arabidopsis* (Llave et al., 2002). However, better opportunities to characterize miRNAs in plants have arisen via high-throughput sequencing platforms, especially in non-model organisms for which the full genome sequence has yet to be acquired, such as *Cunninghamia lanceolata* (Wan et al., 2012), *Rehmannia glutinosa* (Yang et al., 2011a), *Citrusre ticulata* (Guo et al., 2016), and *Asparagus officinalis* (Chen et al., 2016). Moreover, cleaved miRNA targets can also be identified by degradome sequencing at the transcriptome level, overcoming the problem that predicted target genes must otherwise be independently validated (Addo-Quaye et al., 2008; Li et al., 2010). To examine the differential accumulation of miRNAs that respond to *B*. *elliptica*, three libraries of sRNAs from *L. regale* under *B. elliptica*-mock treatment and *elliptica*-infection for 6 and 24 h were constructed, which were sequenced using the Solexa/Illumina platform with the transcriptome of *L. regale*. In addition, the corresponding target genes of these miRNAs were also identified by degradome sequencing in lily. The expression patterns of miRNAs under *B. elliptica* infection and their targets were identified and further verified by quantitative real-time PCR (qRT-PCR). The results suggest the presence of regulatory miRNAs in the economically important ornamental plant lily, and also shed new light on the miRNA-mediated regulatory networks that respond to fungal infection in lily.

## Materials and methods

### Plants and *B. elliptica* inoculation

The *B. elliptica*-resistant *Lilium regale* Wilson and the *B. elliptica*-susceptible Asian hybrid “Tresor” as host plants were planted on culture medium under a 12 h day/night cycle at 25/22°C and placed in a greenhouse at Beijing Forestry University. The plants for treatment were conducted during the flower bud stage. *Botrytis elliptica* isolated from diseased lily leaves was grown on potato dextrose agar medium under near-UV light for 7 days. For biotic stress treatment, leaves detached from the fifth to tenth sites below the apex were first carefully washed under distilled water and then surface-sprayed with 5 × 10^4^ conidia·mL^−1^ of *B. elliptica* (Liu et al., 2016). Subsequently, all of the treated leaves were placed in trays covered with preservative film to ensure a relative humidity of 90–100%. When leaf tissues were harvested at 0, 6, 12, and 24 h postinoculation (hpi), sample pools were established from at least five independent plants for each sampling time, The sampling time set from early experiments (Liu et al., 2016) and gene expression variation in the transcriptome sequencing in our laboratory (data not published). All of the collected samples were immediately frozen in liquid nitrogen and stored at −80°C until RNA extraction.

### RNA extraction, small RNA library construction and sequencing

Total RNA from lily leaves of the control and treated samples was extracted using the EASYspin Plus Plant RNA kit (Aidlab Bio, Beijing, China) according to the manufacturer's protocol. Three small RNA libraries of *L. regale* (*B. elliptica*-mock: “BE0h,” *B. elliptica*-infected for 6 h: “BE6h,” *B. elliptica*-infected for 24 h: “BE24h”) were constructed by Solexa/Illumina sequencing (LC Bio, Hangzhou, China). Briefly, the population of small RNAs was initially isolated, purified, and subsequently ligated to Solexa adaptors at each end. Then, the assembled small RNAs were reverse-transcribed to cDNA, followed by PCR, and the purified PCR products were eventually used for cluster generation and sequencing analysis with the ACGT101-miR program (version 4.2; LC Bio). The results were deposited in the Sequence Read Archive (SRA) at NCBI database (accession number: SRP103981).

### Analysis of sRNA sequencing data

After the sequencing reactions, the raw reads were first subjected to filtering out adaptor dimers, junk reads and low-quality tags, and then mapped onto transcriptome of *L. regale*. Subsequently, perfectly matched sequences were compared against non-coding RNAs from Rfam database (http://rfam.sanger.ac.uk/) and the NCBI GenBank database (http://www.ncbi.nlm.nih.gov/genbank) to classify the degradation fragments of non-coding RNAs, which were excluded from further analysis. The remaining sequences were aligned with the mature and precursors of miRNAs in miRBase 20.0 (http://mirbase.org) by BlastN to identify conserved miRNAs. Furthermore, the unannotated small RNAs were analyzed for the prediction of novel miRNAs.

### Identification and validation of novel miRNAs of *L. regale*

For novel miRNAs identification, the hairpin RNA structures of novel miRNAs were predicted by RNAfold software and the criteria were accorded as follows: (1) the miRNA and miRNA^*^ are located in opposite stem-arms and form a duplex with two nucleotide 3′ overhangs; (2) the number of mismatched bases between miRNA and miRNA^*^ <4; (3) asymmetric bulges are minimal in size (typically two or less); (4) the potential miRNA precursor with minimal folding energy indexes (MFEI) >0.6 (Jones-Rhoades et al., 2006; Meyers et al., 2008).

Stem-loop RT-PCR was performed to validate the identification novel miRNAs. Total miRNAs were extracted from samples and reverse-transcribed to cDNA using QuantScript RT Kit (Tiangen Bio, Beijing, China) with specific RT primer (Supplementary Table S4). The RT-PCR reaction was continued at 94°C for 3 min, followed by 30 cycles of 95°C for 30 s, 55°C for 30 s, and 72°C for 15 s, ending with 72°C for 5 min with 2 × Taq PCR MasterMix (Aidlbab Bio, Beijing, China). Amplification products were separated with 2.5% agarose gel electrophoresis.

### Identification of *B. elliptica*-responsive miRNAs

To identify *B. elliptica*-responsive miRNAs of *L. regale*, the miRNA abundance in the three libraries was normalized by the total number of miRNAs per sample (Zhang et al., 2016). If the normalized read count of a given miRNA was zero, the expression value was modified to 0.01 for further analysis. The expression levels of miRNAs between stress and control groups (BE6h/BE0h, BE24h/BE0h) were calculated as follows: fold-change = log_2_ (BE6h/BE0h or BE24h/BE0h). When the fold-change with a *p* ≤ 0.01 was more than 2.0 or less than −0.5, the miRNA was considered to be significantly differentially expressed in response to *B. elliptica*. The *p*-value was calculated according to previously established methods (Man et al., 2000).

### Degradome library construction and target identification

To investigate the potential target mRNAs, a degradome library was constructed as previously described (Addo-Quaye et al., 2008). Similar to the small RNA libraries, the degradome cDNA library was sequenced on Illumina GAIIx (LC Bio, Hangzhou, China). After removing adaptor sequences and low quality sequencing reads, the clean reads were used to identify potentially cleaved targets based on *L. regale* assembled transcriptome sequences. Meanwhile, the degradome analysis and the classification of target categories were performed with CleaveLand 3. Moreover, Cytoscape 3.2 software was used to generate and visualize the miRNA-mediated gene regulatory network.

### Quantitative real-time PCR (qPT-PCR) analysis of miRNA expression

The expression of 12 selected miRNAs and 6 targets from the different times postinoculation was assayed using qRT-PCR to validate results obtained from high-throughput sequencing of miRNAs and their targets. Total RNAs and miRNAs were extracted from samples and reverse-transcribed to cDNA using ReverTra Ace® qPCR RT Master Mix with gDNA Remover (Toyobo, Shanghai, China) following the manufacturer's instructions (Chen et al., 2012; Liu et al., 2016). qRT-PCR was performed using a total reaction volume of 20 μL, which contained 2 μL of diluted cDNA, 1 μL primer mix, 10 μL of 2 × qPCR mix, and 7 μL ddH_2_O. Each reaction was performed on a MiniOpticon Real-time PCR Detection System (Bio-Rad, Hercules, CA, USA), using SYBR Premix Ex Taq (Takara, Otsu, Japan) in triplicate. Amplification reactions were performed as follows: 95°5 for 5 s, 69°9 for 20 s, and 72°2 for 20 s. All reactions were performed in triplicate. The *18S rRNA* and *CLATHRIN* genes were used as the reference genes for normalization and the relative expression levels were calculated using the 2^−heCT^ method (Ginzinger, 2002). The primers for amplification were designed using the Beacon Designer 7 software, as listed in Supplementary Table S5.

## Results

### High-throughput sRNAs sequencing in *L. regale* wilson

To reveal *Botrytis elliptica*-responsive microRNAs in lily, three sRNA libraries (BE0h, BE6h, and BE24h) were constructed and sequenced using Illumina Genome Analyzer IIx (LC Bio, Hangzhou, China). Deep sequencing of the libraries generated a total of 26.44 million raw reads representing 3.52 million unique sequences from the three sRNA libraries (Table 1). Based on removal of the low-quality tags and adaptor contaminants, 2,556,296, 6,981,340, and 5,049,471 clean sequences were obtained for these three libraries, respectively, with a size of 17–25 nt. After further filtering out the Rfam (rRNA, tRNA, snRNA, snoRNA, and other Rfam RNAs) and Repbase sequences, the remaining reads were queried against miRBase 20.0 database. From that, totals of 2,065,391 (representing 431,479 unique sequences), 5,981,827 (representing 724,767 unique sequences), and 4,162,571 (representing 587,701 unique sequences) sRNA sequences, respectively, were obtained and used for the identification of conserved and novel miRNAs (Table 1).

**Table 1:** **Distribution of small RNAs among different categories in three libraries**.

**Category**	**BE0h**		**BE6h**		**BE24h**	
	**Total**	**Unique**	**Total**	**Unique**	**Total**	**Unique**
Raw reads	8,401,329	941,574	10,009,977	1,344,881	8,029,349	1,231,079
3ADT & length filter	5,839,180	465,531	3,013,669	551,840	2,969,128	574,094
Junk reads	5,853	3,549	14,968	8,562	10,750	6,336
Rfam	485,876	39,348	990,789	57,110	875,868	60,392
Repeats	6,243	1,863	14,144	2,982	16,211	3,013
rRNA	216,044	26,282	515,833	36,808	489,412	36,989
tRNA	225,325	8,025	338,444	11,798	280,879	14,854
snoRNA	5,546	976	29,390	1,620	21,651	1,764
snRNA	4,264	989	6,413	1,382	5,884	1,399
Other Rfam RNA	34,697	3,076	100,709	5,502	78,042	5,386
Clean reads	2,065,391	431,479	5,981,827	724,767	4,162,569	587,701

**Figure 1:**
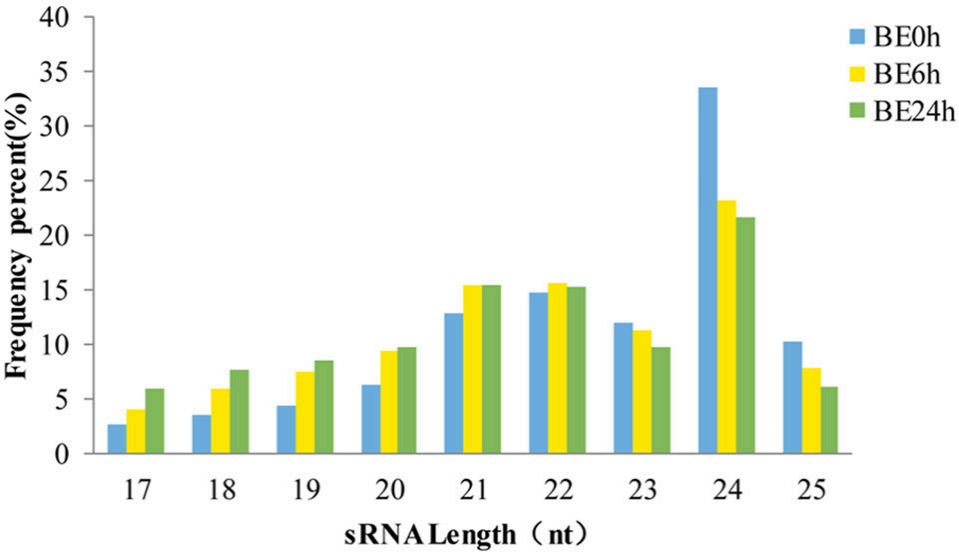
**Length distribution of small RNAs sequences in three libraries**.

The length distributions of the sRNA sequences, which ranged from 17 to 25 nt, were similar among the three libraries (Figure 1), with 24 nt representing the most common length, and followed by 21 and 22 nt. These results are consistent with the observed length distribution of mature plant miRNAs, such as in *Arabidopsis thaliana* (Sunkar and Zhu, 2004), *Solanum lycopersicum* (Jin and Wu, 2015), and *Raphanus sativus* L. (Wang Y. et al., 2015; Figure 1). Specifically, the relative abundances of 24-nt sRNAs in the BE6h and BE24h groups were markedly lower than those in the *elliptica*-mock library, suggesting that the 24-nt miRNA classes might be repressed under *B. elliptica* infection. Conversely, both 21- and 22-nt sequences in the BE6h and BE24h were notably more abundant than those in BE0h library. Thus, the sRNAs with different sizes probably perform different functions: 21-nt sRNAs usually mediate gene silencing at the post-transcriptional level, while 24-nt sRNAs typically perform gene silencing mediated by RNA-dependent DNA methylation and heterochromatin maintenance (Duan et al., 2016).

### Identification of known miRNAs in *L. regale* wilson

**Figure 2:**
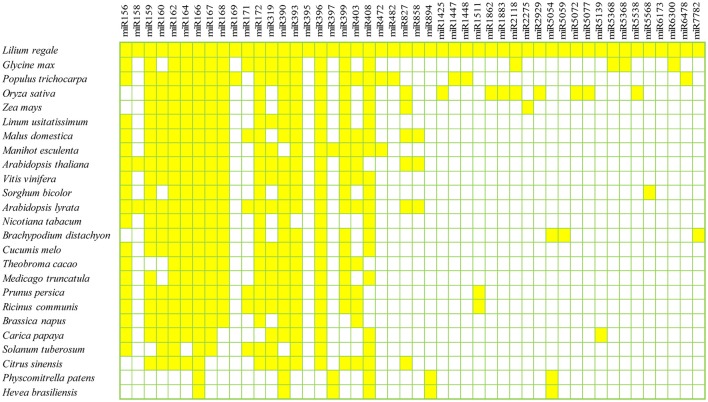
**Conserved miRNA families in ***L. regale*** and across species**. All miRNAs of *L. regale* were identified based on sRNA sequencing data, and those of other plants were obtained from miRBase 20.0.

**Figure 3:**
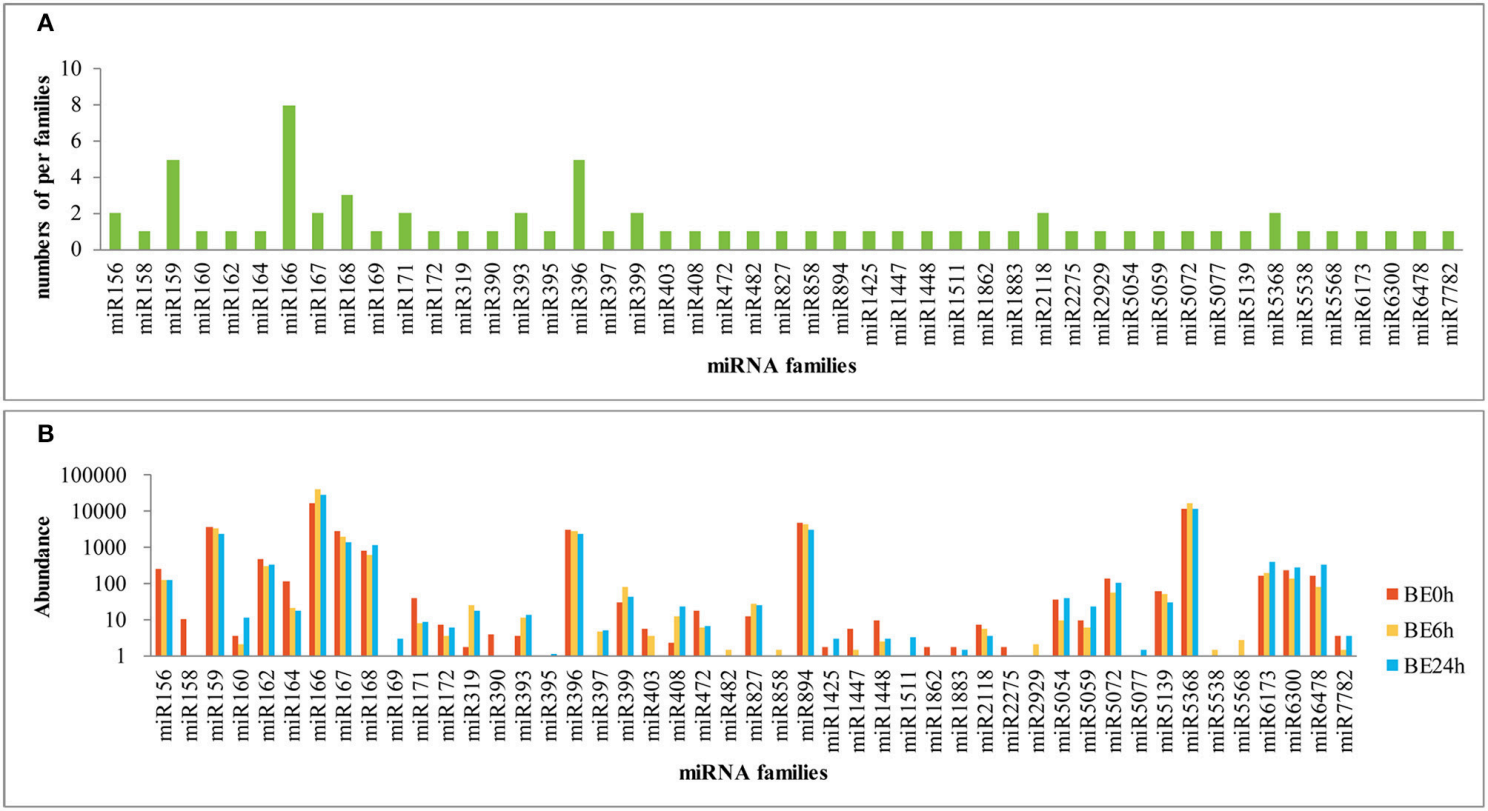
**Number and abundance of identified known miRNA families from lily (A:** distribution of known miRNA family members in lily; **B**: counts of each known miRNA family in lily).

To identify known miRNAs in the three libraries, the clean reads were compared with known miRNA precursor or mature miRNA sequences in miRBase 20.0, allowing no more than two mismatches. Ultimately, 71 non-redundant miRNAs belonging to 47 conserved families were identified in the three libraries (Supplementary Table S1), which showed high sequence similarity to those of other plant species in miRBase (Figure 2). Among the 47 miRNA families, the numbers of the conserved miRNA family members were dramatically different (Supplementary Table S1 and Figure 3A). For example, the miR166 family was the most highly represented with eight members, followed by miR159 and miR396 with five members each. Of the remaining miRNA families, most possessed a single member, although these families are known to contain more members in other plants (e.g., miR158, miR171, miR172, and miR319). Moreover, unique sequences, found in few other plant species, were detected in the lily libraries. For instance, miR5072 has only been registered in *Oryza sativa*, and miR5054 in *Brachypodium distachyon* (Schreiber et al., 2011).

The relative abundance of different conserved miRNA families can be estimated by the number of reads, which exhibited large divergences. A few conserved miRNA families, such as miR159, miR166, miR167, miR396, miR894, and miR5368, showed extraordinarily high expression abundance among the three libraries, of which the miR166 family exhibited the most abundant expression with reads accounting for over 50% of all conserved miRNA reads (Figure 3B). Moreover, miR162, miR164, miR827, miR5072, miR6173, miR6300, and miR6478 were moderately abundant in the three libraries, although they contained only a single member each. However, a number of miRNA families, such as miR395, miR482, miR858, and miR1883 showed relatively low expression levels, with no more than five reads in the three libraries (Figure 3B). The abundance of miRNA families can dramatically vary, suggesting functional variation within each family. In addition, distinct members of the same miRNA family also showed dramatically different levels of expression. For instance, the abundance of miR166 members varied from 0 to 8,911 reads in the BE0h library (Supplementary Table S1), likely due to function-specific expression of different miRNA members (Jiang et al., 2006).

### Prediction of novel miRNAs in *L. regale* wilson

**Figure 4:**
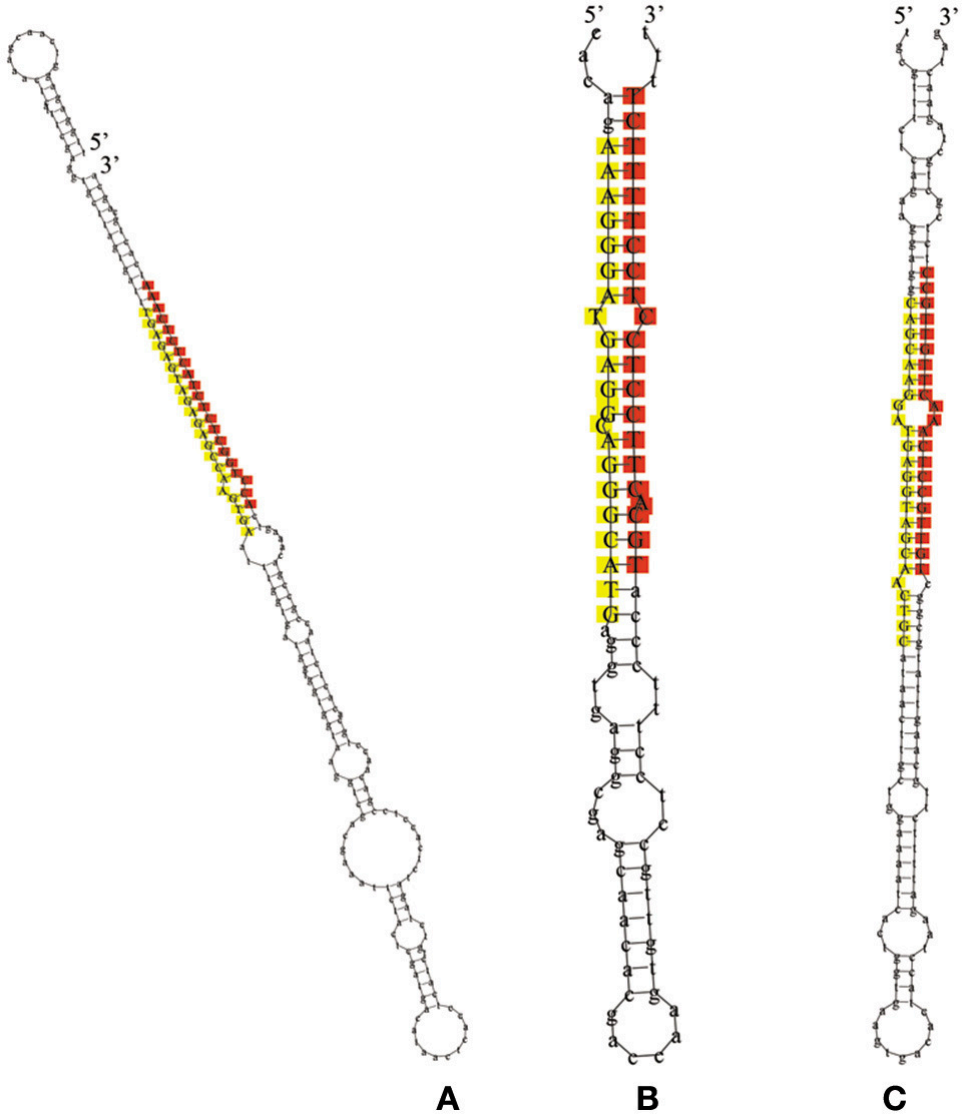
**The hairpin structures for precursors of novel miRNAs**. The red colored sequences represent mature miRNAs, and the yellow colored sequences represent the miRNA^*^ (**A** represents miRn3, **B** represents miRn4, and **C** represents miRn5).

The remaining reads that had not been mapped to selected miRNAs in miRBase, but perfectly matched to the transcriptome of *L. regale*, were identified as potentially novel miRNAs, of which the precursors with canonical stem-loop structures were further analyzed using a series of stringent filter strategies to ensure that they satisfied the common criteria for annotation of plant miRNAs established by the research community (Jones-Rhoades et al., 2006; Meyers et al., 2008). Based on these criteria, 24 novel miRNA sequences were identified, of which only five were determined to contain complementary miRNA^*^s (Supplementary Table S2). Because of the instability of miRNA^*^ in cells, miRNAs without miRNA^*^s were also regarded as novel miRNAs. The length of mature novel miRNAs varied from 17 to 25 nt, with the majority being 21 nt, and their precursors ranged from 55 to 282 nt with a mean length 151 nt (Supplementary Table S2). The hairpin structures of the precursors of miRn3, miRn4, and miRn5 are presented in Figure 4. The values of minimum folding free energy (MFE) of these predicted pre-miRNAs ranged from −185.8 to −19 kcal mol^−1^ (Supplementary Table S2). Consistent with the characteristics of miRNAs, the MFEI ranged from 0.6 to 3. The expression levels of the novel miRNAs varied significantly in the three libraries (Supplementary Table S2). Although most of the novel miRNAs had relatively low expression, these miRNAs might be specific to lily. To validate these novel miRNAs, stem-loop RT-PCR was performed in miRn3, miRn5, miRn22, and miRn24 as examples. As shown in Supplementary Figure S1, these novel miRNAs had signal on agarose gels, indicating that they can be considered as novel miRNAs of lily.

### miRNAs differentially expressed in response to *B. elliptica*

**Figure 5:**
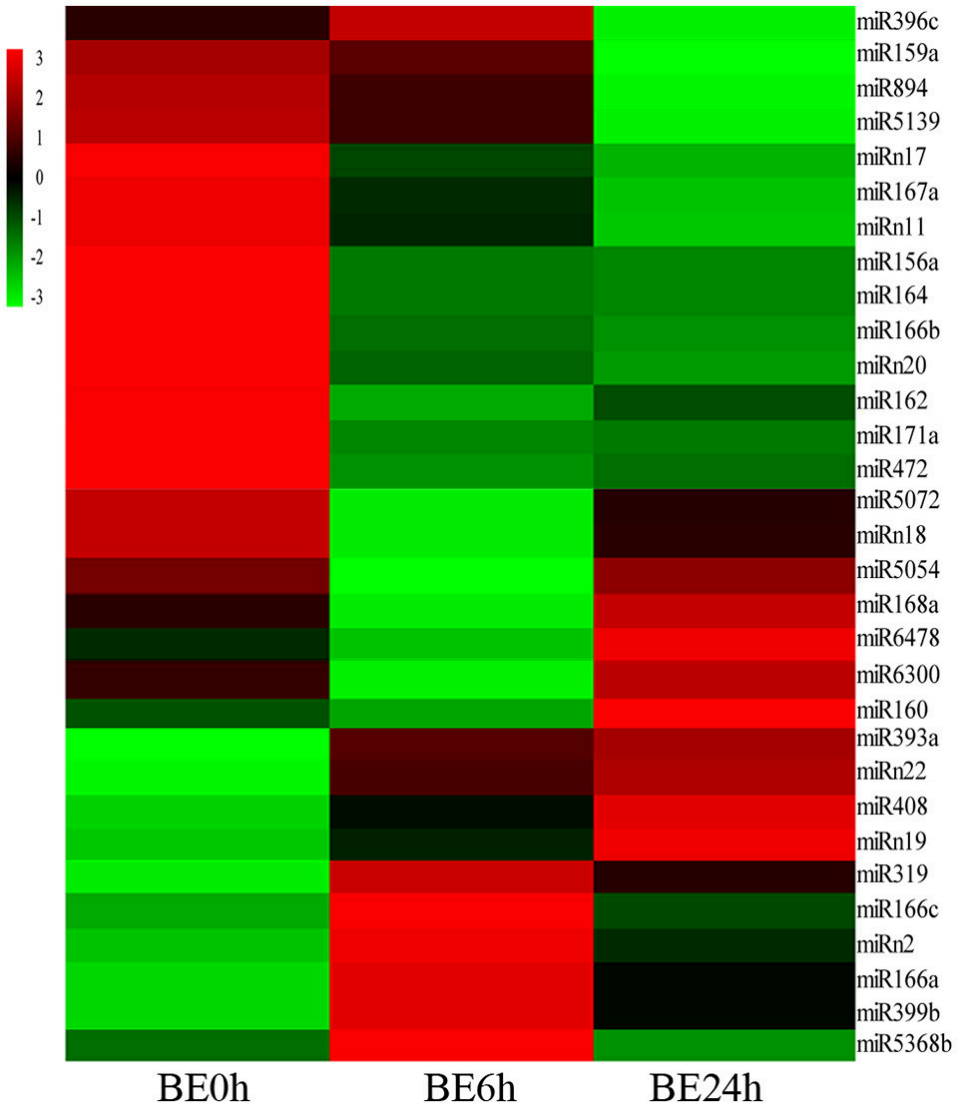
**Heatmaps show the differential expression of 24 conserved and 7 novel miRNAs of ***L. regale*** under ***B. elliptica*** infection 0, 6, and 24 h with ***p*** ≤ 0.01**. Red indicates higher levels of miRNAs and green indicates lower levels. The absolute signal intensity ranges from –3.0 to +3.0, with corresponding color changes from green to red.

To systematically identify *B. elliptica*-responsive miRNAs in *L. regale*, a normalized differential expression pattern analysis was performed between the *B. elliptica*-mock library and the two *B. elliptica*-infected libraries. Among all of the identified conserved and novel miRNAs, 24 conserved and 7 novel miRNAs were identified to be significantly differentially expressed upon comparing these three libraries (*p* ≤ 0.01, log_2_Ratio > 2 or < −0.5; Figure 5, Supplementary Table S3). Of these, 8 known miRNAs, namely, miR160, miR166a, miR166c, miR319, miR393a, miR399b, miR408, miR5368b, and 3 novel miRNAs were upregulated continuously in *elliptica*-infected leaves (BE6h and BE24h) compared with their levels in *elliptica*-mock (BE0h); in contrast, 13 known and 4 novel miRNAs were downregulated in BE6h and BE24h (Figure 5). Intriguingly, two miRNAs (miR168a and miR6478) firstly declined in BE6h and then peaked in BE24h; while miR396c exhibited the opposite trend. Among all of the markedly differentially expressed miRNAs, miRNA319 was considered to be the most significantly upregulated known miRNA, with a 3.85-fold increase of expression (BE6h/BE0h), while miR164 was the most downregulated. We found that the most differentially expressed miRNAs were downregulated under *B. elliptica* treatment. These significant variations in miRNA expression implied that these miRNAs could play crucial roles in the response to *B. elliptica* of lily.

### Identification of the *B. elliptica*-responsive miRNA targets by using degradome analysis

**Figure 6:**
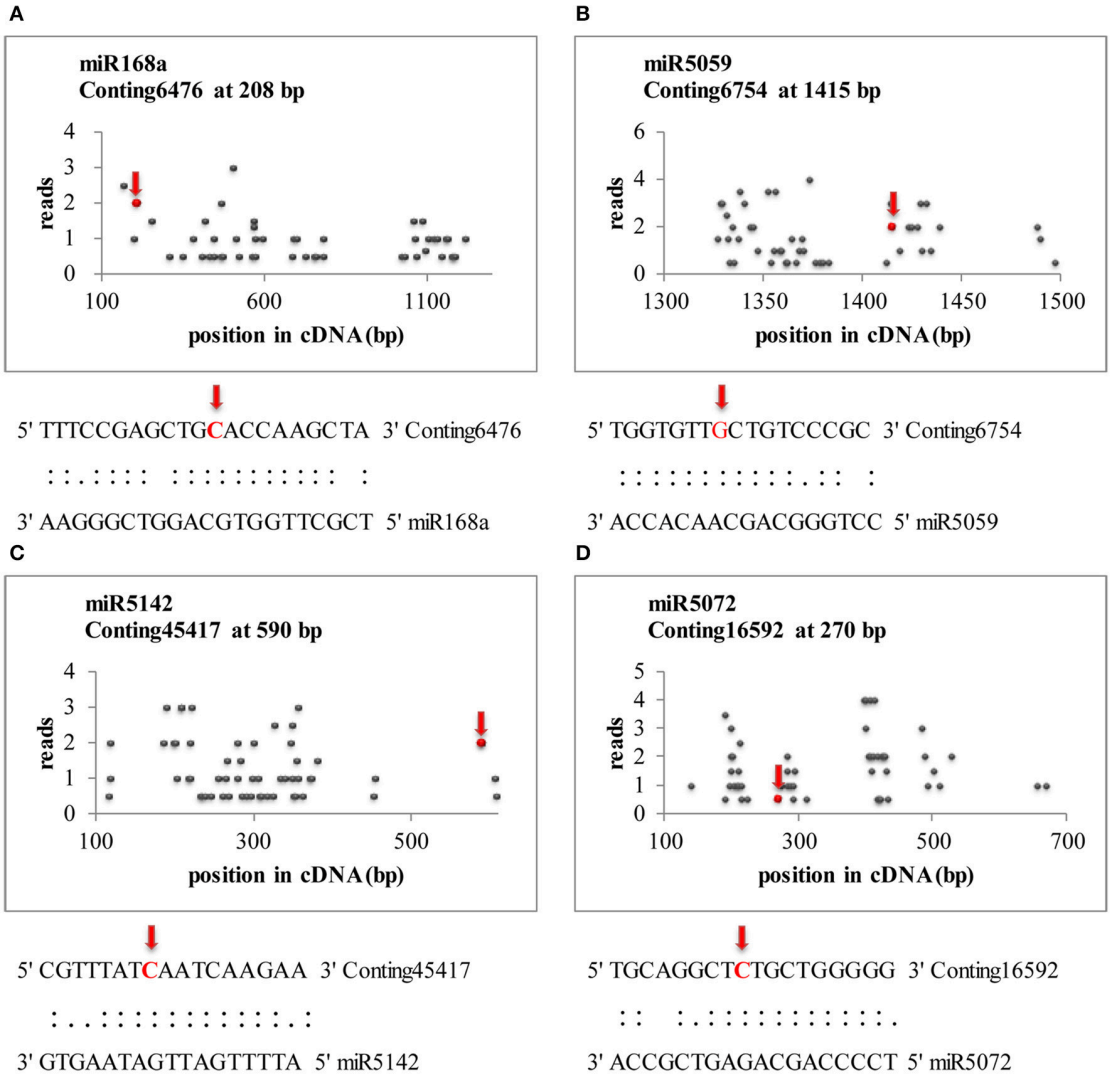
**Target plots (***t***-plots) of miRNA targets identified by degradome sequencing in lily**. Red arrows represent the nucleotide position of cleavage on the target genes. **(A–D)** Represents miR168a, miR5059, miR5142, and miR5072, respectively.

Transcriptome-wide analysis of the miRNA-cleaved mRNAs using degradome sequencing technology was performed to understand the biological function of miRNAs in lily. After discarding the low-quality sequences, a total of 13,026,025 raw reads with 3,146,014 unique raw reads were remained. Then, 5,550,212 of the sequences were mapped to the *L. regale* transcriptome to identify the fragments of degraded mRNAs. In total, 22 target transcript sequences that could potentially be cleaved by 10 miRNAs (including 9 known and 1 novel ones) were identified through the Cleveland pipeline (Figure 6, Table 2). Generally, several targets were regulated by only a single miRNA. For instance, miR166b and miR5059 had the most target genes (miR166b targeted five genes and miR5059 targeted seven genes), indicating that these two families might play diverse roles in resistance to *B. elliptica* stress. Furthermore, six known miRNAs (miR319, miR408, miR1862, miR2275, miR5072, and miR5142) had only one target each.

**Table 2:** **Identified target transcripts for the known and novel miRNAs in lily**.

**miR name**	**miR sequence**	**Target number**	**Annotation**
miR166b	GGAATGTTGTCTGGCTCG	gi421958342	Unknown
	GGAATGTTGTCTGGCTCG	Conting1715	Elongation factor 1-alpha 1
	GGAATGTTGTCTGGCTCG	gi73944880	Unknown
	GGAATGTTGTCTGGCTCG	Conting1692	Elongation factor 1-alpha 1
	GGAATGTTGTCTGGCTCG	Conting39906	Sodium/hydrogen exchanger 6
miR168a	TCGCTTGGTGCAGGTCGGGAA	Conting6476	Hypothetical protein
	TCGCTTGGTGCAGGTCGGGAA	Conting6477	Hypothetical protein
miR319	TTTGGACTGAAGGGAGCTCCT	Conting4725	Transcription factor TCP2
miR408	TGCACTGCCTCTTCCCTGGCT	Conting23422	Unknown
miR1862	ACTAGATTTGTTTATTT	Conting15313	Agmatine deiminase
miR2275	TTCAATTTCCTCTAATATC	gi262091961	Ubiquitin-conjugating enzyme 2
miR5059	CCTGGGCAGCAACACCA	Conting5555	Heat shock protein
	CCTGGGCAGCAACACCA	Conting5580	Heat shock cognate 70 kDa protein 2
	CCTGGGCAGCAACACCA	Conting6754	Hydroxycinnamoyl-Coenzyme A shikimate/quinate hydroxycinnamoyltransferase
	CCTGGGCAGCAACACCA	Conting332	Copper chaperone
	CCTGGGCAGCAACACCA	Conting1004	12-oxophytodienoate reductase 3
	CCTGGGCAGCAACACCA	Conting13784	Unknown
	CCTGGGCAGCAACACCA	Conting6009	Protein kinase G11A
miR5072	TCCCCAGCAGAGTCGCCA	Conting16592	Hypothetical protein
miR5142	ATTTTGATTGATAAGTG	Conting45417	Uncharacterized protein
miRn24	ACATAATCATGATCTTCC	Conting11288	Predicted protein
	ACATAATCATGATCTTCC	Conting5180	Vesicle-associated membrane protein

The target genes of miRNAs were classified in a centralized functional distribution. Most genes were involved in metabolism, including diverse metabolic enzymes such as *12-OXOPHYTODIENOATE REDUCTASE 3* (*OPR3*) and *AGMATINE DEIMINASE* (*ADI*), which were cleaved by miR5059 and miR1862, respectively (Table 2). Besides, two target genes, encoding the transcription factors *EF1a* and *TCP2*, were targeted by miR166b and miR319 (Table 2). These transcription factors could participate in various aspects of plant development and stress responses and also act as the main nodes in gene expression networks in plants. *HEAT SHOCK PROTEIN* (*HSP*), which could be involved in response to biotic and abiotic stresses, was a target gene of miR5059. Generally, most of the targets were involved in various types of signal sensing and transduction, such as reactive oxidative species (ROS) signaling, the biosynthesis of jasmonic acid (JA), and cell wall synthesis, which were considered to be involved in the response to *B. elliptica*. Although sequencing revealed that the novel miRNAs were present at a lower expression level than the known miRNAs, their targets were also identified by degradome sequencing analysis. However, unknown, hypothetical, or predicted proteins and even no targets were identified for other miRNAs in the degradome sequencing data, partly due to the limited number of accessible lily reference sequences.

### Validation of *B. elliptica*-responsive miRNAs and their targets by qRT-PCR

**Figure 7:**
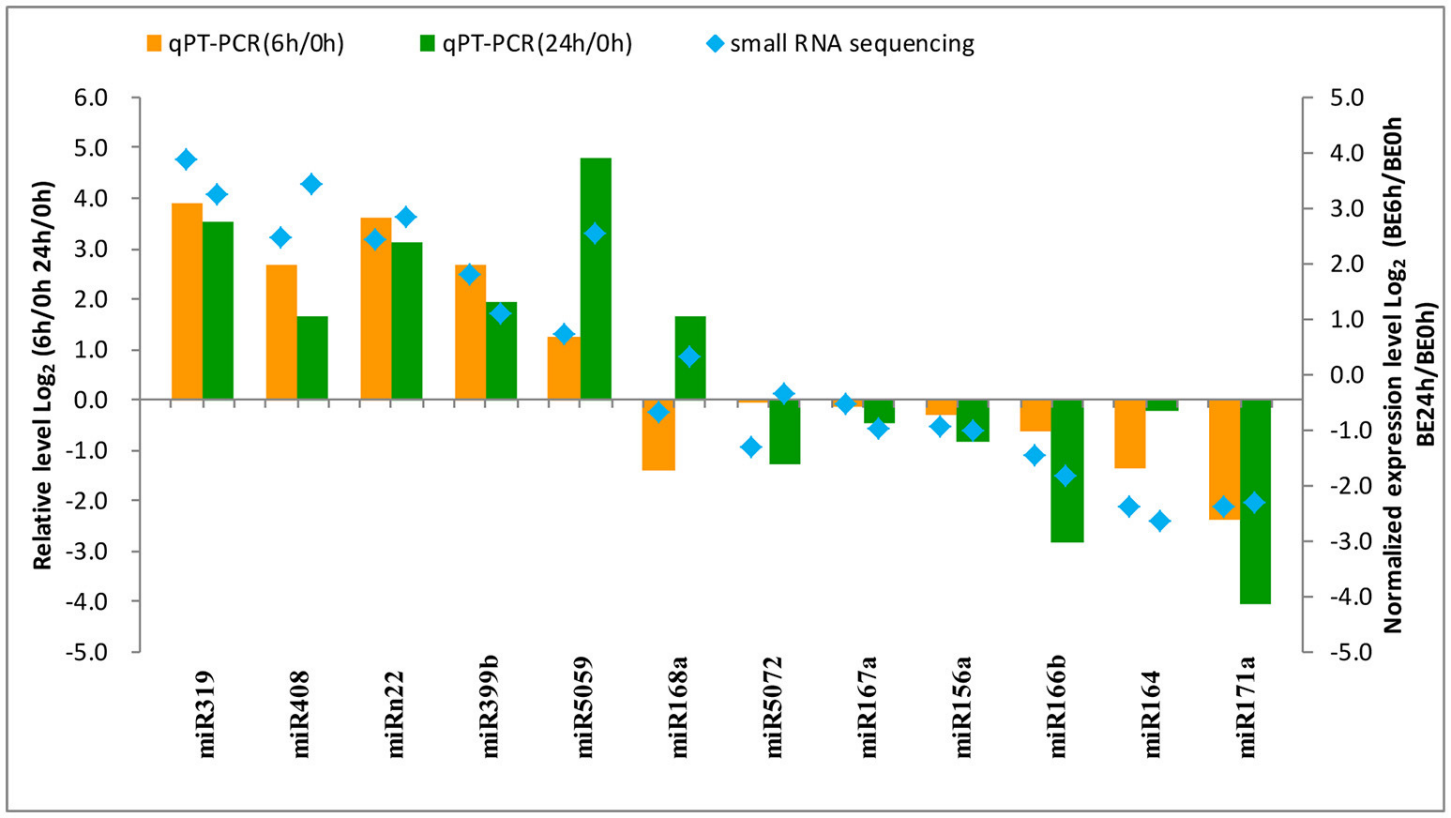
**Analysis of the differential expression of miRNAs with high abundance by comparing the deep sequencing (right ***y***-axis) and qRT-PCR (left ***y***-axis) in ***L. regale*****. The fold-change of miRNAs was transformed as Log_2_ (treat/CK) from date of the deep sequencing and qRT-PCR.

To confirm the results obtained from small RNA deep sequencing, quantitative real-time PCR was performed to confirm the expression levels of 12 miRNAs among the different times of *B. elliptica* infection of *L. regale*. As shown in Figure 7, most of the expression patterns of *B. elliptica*-responsive miRNAs from qRT-PCR in *L. regale* were similar to those obtained by deep sequencing. However, the fold-change of expression obtained by qRT-PCR was not completely consistent with the results of bioinformatic analysis, which partially due to the differences in the sensitivity, specificity, and algorithm between the two techniques.

**Figure 8:**
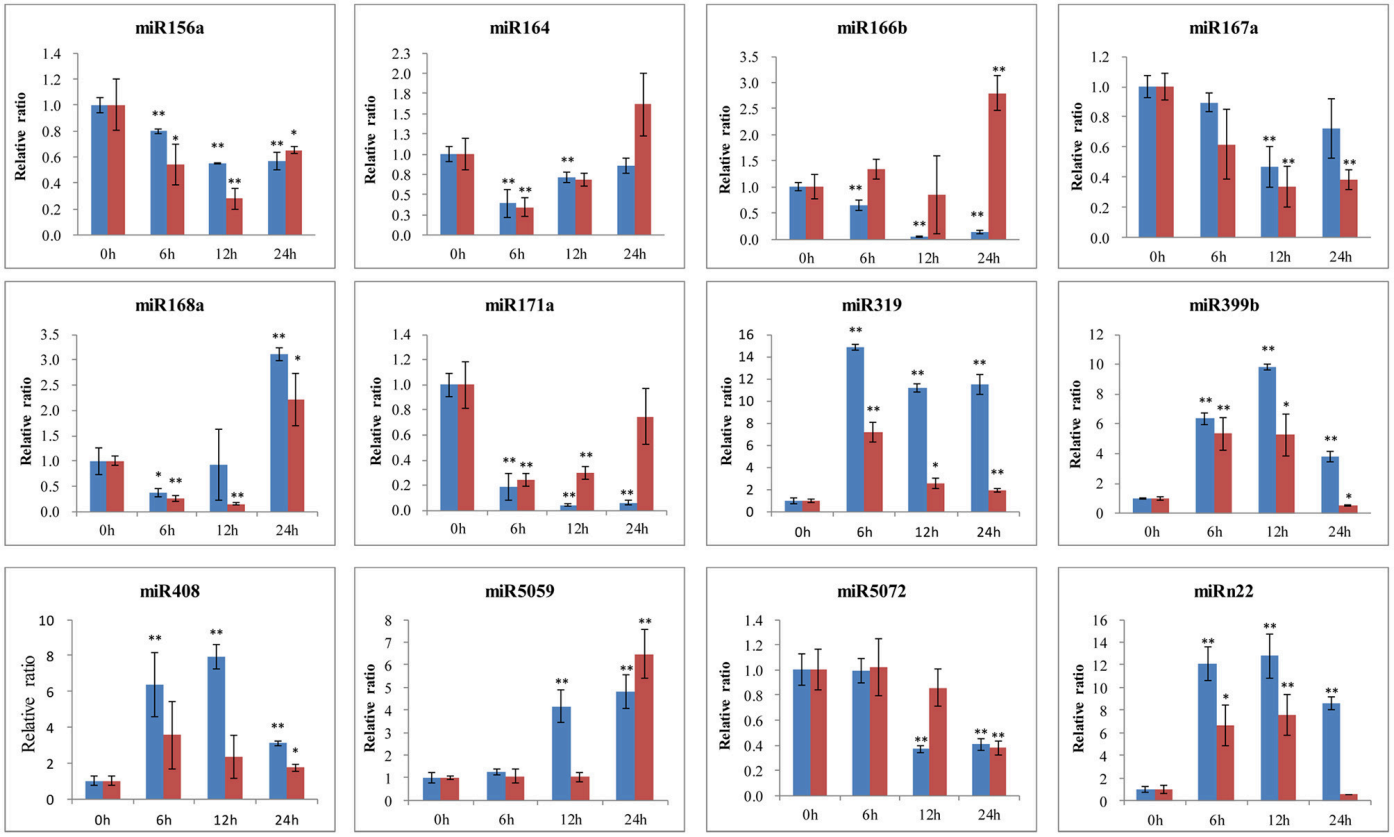
**qRT-PCR validation of ***B. elliptica***-responsive miRNAs in lily**. The blue bar represents the resistant *L. regale*, and the red bar represents the susceptible “Tresor.” The amount of expression was normalized to the level of *18S rRNA*. The normalized miRNA levels at 0 h were arbitrarily set to 1. Each bar shows the mean ± *SE* of triplicate assays. ^*^ or ^**^ indicates a statistically significant difference as relative to the value at 0 h for each miRNAs at *p* < 0.05 or 0.01, respectively.

To further identify the function of conserved and novel miRNAs in lily, the expression levels were detected by qRT-PCR in both the *B. elliptica*-resistant *L. regale* and the *B. elliptica*-susceptible Asian hybrid “Tresor.” As shown in Figure 8, the expression of most miRNAs seems to share the same trend in the two species during pathogen attack and much lower expression levels were detected in the susceptible cultivar “Tresor.” Of these, five miRNAs (miR319, miR399b, miR408, miR5059, and miRn22) were upregulated and peaked at 6 or 12 h in both the two species. Another three miRNAs (miR156a, miR167a, and miR5072) had a downregulated expression pattern. Meanwhile, three miRNAs seem to show different expression patterns of the two species with different resistant to *B. elliptica*. For example, the level of miR164 transcripts sharply dropped at 6 h, and then continued at a low level in *L. regale*. However, the expression of miR164 was raised at 24 h in “Tresor.” Besides, miR166b and miR171a were exhibited an opposite expression profile in the two species. In the resistant *L. regale*, miR166b was downregulated after *B. elliptica* infection; to the contrary, miR166b was gradually upregulated at 24 h in “Tresor.” These differently expressed miRNAs might play key roles in resistant of *B. elliptica*.

**Figure 9:**
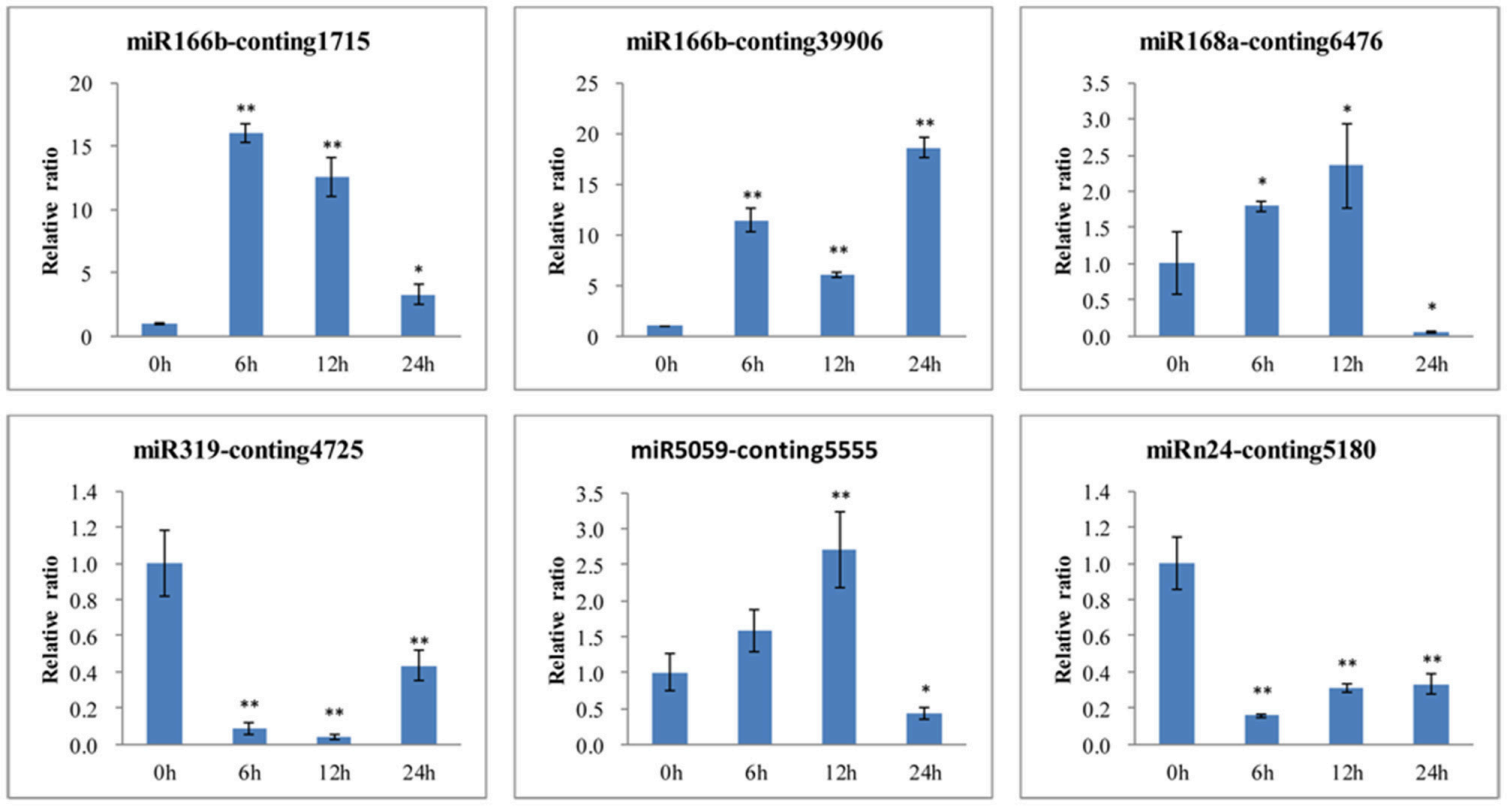
**qRT-PCR validation of ***B. elliptica***-responsive miRNA target genes in ***L. regale*** (Conting 1715, Conting 39906, Conting 5555, Conting 6476, Conting 4725, and Conting 5180 represent genes encoding ***EF1***, ***SODIUM/HYDROGEN EXCHANGER 6***, ***HSP***, ***HYPOTHETICAL PROTEIN 1***, ***TCP2***, and ***VESICLE-ASSOCIATED MEMBRANEPROTEIN***)**. The amount of expression was normalized to the expression level of the *CLATHRIN* gene. The normalized mRNA levels at 0 h were arbitrarily set to 1. Each bar shows the mean ± *SE* of triplicate assays. ^*^ or ^**^ indicates a statistically significant difference as relative to the value at 0 h for each miRNAs at *p* < 0.05 or 0.01, respectively.

To further confirm the dynamic correlation between the miRNAs and their corresponding targets, the expression patterns of six target genes were examined by qRT-PCR of *L. regale* at different time points of *B*. *elliptica* infection (Figure 9). As expected, an approximately negative correlation was observed between miRNAs and their targets. For example, *EF1a* and *NHE-6* (Conting 1715 and Conting 39906), two targets of miR166b, were upregulated at 6 h; whereas miR166b sharply declined at that time. Moreover, the expression level of *TCP2* was significantly reduced at 6 h, the time at which the expression of miR319 was relatively enhanced. It could be found that the relative expression trends between the *B. elliptica*-responsive miRNAs and their corresponding targets were generally inversed.

## Discussion

### miRNA library construction of *L. regale* wilson

miRNAs, as key factors in numerous cellular events, play multiple roles in regulating many biological processes in plants. With the development of high-throughput sequencing and bioinformatic approaches, miRNAs have been identified from various plant species without fully sequenced genomes, including *Hemerocallis fulva* (An et al., 2014), *Phalaenopsis Aphrodite* (An and Tsair, 2012), *Asparagus officinalis* (Chen et al., 2016), and *Rehmannia glutinosa* (Yang et al., 2011b). Little research has been conducted to identify miRNAs from *L. regale*, because of the limited genomic data which restricts sRNAs annotation and identification of the many sources of *Lilium* sRNA production. In this study, we conducted the first screen for *L. regale* miRNAs by deep sequencing with three *B. elliptica*-infection libraries. To increase the accuracy, an mRNA transcriptome from *B. elliptica*-infected *L. regale* provided by our laboratory was also used as a reference sequence for miRNA high-throughput sequencing and could provide more valuable information for degradome sequences analyses. Moreover, the expression pattern of transcriptome mRNAs could also be a reference of miRNA-target gene interaction.

This research resulted in the characterization of 71 known miRNAs across 47 conserved families in lily. Most miRNA families identified in lily have also been found in other model plants. For example, the sequence of lre-miR166 family had a perfect match with miR166 in other species, such as *Glycine max, Populus trichocarpa* and *Oryza sativa* (Figure 2). This implies that the miRNAs are well-developed in both monocotyledons and dicotyledons, and might play the similar roles in their growth and development. Moreover, some miRNAs, that had been identified in just a few species, even in only one other species, were found in *L. regale*. For example, miR5059 and miR6300, which have to date only been registered in *Brachypodium distachyum* (Schreiber et al., 2011) and *Glycine max* (Turner et al., 2011), also appeared in lily. Furthermore, 24 novel miRNAs with low abundance were discovered in *Lilium*, and these miRNAs with limited expression might be specifically involved in the response to *B. elliptica* in lily.

### Characteristics of *B. elliptica*-responsive miRNAs in lily

Solexa sequencing not only provides the foundation for identifying known and novel miRNAs, but is also a way of estimating the expression profiles of miRNA (Wang et al., 2009). Here, via high-throughput sequencing, we found that 24 known and 7 novel miRNAs were significantly responsive to *B. elliptica* infection compared with their levels in the uninfected control. Among the differentially expressed miRNAs, the majority were downregulated under diseases stress, which is in accordance with findings in other species suffering biotic stress (Luo et al., 2015; Wang and Luan, 2015). For instance, including miR164, miR166, and miR168, the expression of these miRNAs were induced under *Verticillium longisporum* infection in *Brassica napus* (Shen et al., 2014), which were exhibit the same expression pattern in lily under *B. elliptica*-infection. Moreover, other miRNAs could be specifically regulated by *B. elliptica*, mainly *Lilium*-specific ones, including miR5059, miR5139, miR6300, and novel miRNAs, which have rarely been reported in other species. On the other hand, several previously reported disease-responsive miRNAs, such as miR394 in tomato (Jin et al., 2012; Jin and Wu, 2015), were not detected in the current study, which suggested that these miRNAs might be species-specific and that their expression was not specifically altered in lily, or perhaps these miRNAs did not exhibit altered expression during the duration of the study.

In order to further investigate the roles of miRNAs in response to *B. elliptica, L. regale*, and “Tresor” with different levels of resistance to this pathogen were processed for qRT-PCR. It was observed that the higher levels of pathogen-induced expression in resistant plants compared with the pathogen infected susceptible plants, suggesting overexpression of miRNAs might enhance the resistance to *B. elliptica*. For example, miR319 was upregulated eight times higher at 6 h in the resistant *L. regale* than in the susceptible “Tresor.” Previous reports revealed that miR319 was involved in plant responses to drought, salinity, and biotic stresses (Glazov et al., 2008; Naqvi et al., 2010; Jin and Wu, 2015). The different expression level of miR319 between the resistant and susceptible lily suggested that this miRNA might be responsive to *B. elliptica*. Furthermore, miR166 and miR171a were detected to show different expression trend after inoculation between the *B. elliptica*-resistant *L. regale* and the *B. elliptica*-susceptible “Tresor.” It has been reported that miR171 was regulated leaf formations and development (Long et al., 2010). In tomato, miR171a was also detected to be downregulated in response to *B. cinerea* through microarray analysis (Jin et al., 2012). The expression level of miR171 was low possibly due to stress from *B. elliptica* infection to leaves. These results implied that the expression of miRNAs was significantly different because of the plant adaptation and the resistance to *B. elliptica*. However, there is still a need for further studies involving deep profiling of the differential expression patterns and to confirm the precise regulatory roles of these *B. elliptica*-responsive miRNAs in lily.

### Role of miRNAs-target gene mediated gene expression involved in plant defense

To further explore the function of miRNAs in defense against *B. elliptica*, a transcriptome-wide analysis of the degradome was performed, and the target transcripts for the known and novel miRNAs were determined. Through this approach, some miRNAs were easily detectable, but their targets had not been identified yet. Thus, we applied strict selection criteria and identified only 22 targets of these miRNAs by degradome analysis (Table 2). These targets were shown to be involved in signal transduction and plant responses to disease, including transcription factors, enzymes, or proteins in signal pathways, which were regulated by miRNAs through transcript cleavage or translational repression (Sunkar and Zhu, 2004). In our study, it was shown that *TCP2* was likely targets of miR319, which was upregulated after *B. elliptica* inoculation as indicated by decrease of its targets *TCP* transcript, the same as in other reports (Jin and Wu, 2015). As a transcription factor, *TCP2* could most likely downregulated the expression of the ubiquitin proteolytic pathway, which is extensively involved in plant growth, development, and signal transduction of many physiological and biochemical responses (Debigaré and Price, 2003; Yang et al., 2013). Moreover, several studies have shown that *TCP TFs* could bind the TCP-recognized motif (GGACCAC) in the promoter of *LIPOXYGENASE* (*LOX*), which is an enzyme involved in JA biosynthetic pathway (Maksymiec et al., 2005; Schommer et al., 2008). Generally, JAs as endogenous hormones can respond to various biotic and abiotic stresses, which have already been reported in *Arabidopsis* (McConn et al., 1997) and *Vitis* (Jia et al., 2016). Furthermore, *OPR3*, which also controls one of the most important steps of JA biosynthesis (Chen et al., 2006), was identified as a target of miR5059 and upregulated upon 6 h *B. elliptica* infection. These results implied that disease could activate the biosynthesis and accumulation of JA (Zhao et al., 2003), and the JA signaling pathway might increase plant resistance against *Botrytis* (Jia et al., 2016). Another main target of miR5059 was *COPPER CHAPERONE* (*CCH*), a scavenger enzyme of ROS detoxifying superoxide radicals (Beauclair et al., 2010). Downregulation of this miRNA resulted in an increase in *CCH* expression, which could enhance the tolerance to oxidative stress and reduce the damage to plants. *E2*, targeted by miR2275 in lily, which was also targeted by miR399 in rice, could participate in inorganic phosphate (Pi)-signaling pathways, and Pi is a common limiting factor for plant growth (Bari et al., 2006). Several miRNA targets could encode proteins involved in responses to environmental stress. For instance, the *HSP* family is one of the largest groups of proteins induced by heat shock stress; these proteins are present in almost all organisms and have significant functions in cellular homeostasis under adverse environmental conditions (Njemini et al., 2004). In the present study, miR5059 could regulate the expression of *HSPs*, which were also previously reported to be targeted by miR2616 in *Medicago sativa* (Shu et al., 2016) and miR2592 in *Raphanus sativus* (Wang R. et al., 2015), showing that the expression of *HSPs* could be regulated by *B. elliptica* infection. In summary, target genes mediated by miRNAs, which could participate in JA biosynthetic and ROS pathway, enhance the level of defense or tolerance to *B. elliptica* in *L. regale*.

## Conclusion

In conclusion, to the best of our knowledge, this is the first study to perform transcriptome-wide identification of *elliptica*-responsive miRNAs and their targets using small RNA sequencing and degradome analysis in lily. A total of 71 miRNAs, belonging to 47 miRNA families, were identified in *L. regale* leaves subjected to mock *B. elliptica* treatment and *B. elliptica* infection. Among these, 31 miRNAs were differentially expressed in the *elliptica*-infected leaves and responsive to *B. elliptica* stress. Using degradome sequencing, 22 targets cleaved by miRNAs were confirmed in lily. These targets were functionally predicted to transcription factors, biosynthesis metabolic enzymes, biotic, and abiotic stress-responsive proteins. The expression patterns of these differentially regulated miRNAs and their targets were shown to be regulated by *B. elliptica* infection. These results could provide new information for the further identification and characterization of miRNAs in lily, and advance our understanding of the function of miRNAs and their targets in regulating plant responses to *B. elliptica*.

## Author contributions

XG, QL, and GJ designed experiments; XG, QC, and DZ carried out experiments and analyzed experimental results. XG and QzC wrote the manuscript. XG and HH modified the manuscript.

## Funding

This work was supported by National High Technology Research and Development Program of China (863 Program) (Grant No. 2013AA102706) and the National Natural Science Foundation of China (Grant No. 31470106).

### Conflict of interest statement

The authors declare that the research was conducted in the absence of any commercial or financial relationships that could be construed as a potential conflict of interest.

## Supplementary material

Supplementary Figure S1 **Stem-loop RT-PCR electrophoresis of novel miRNAs**.

Supplementary Table S1 **Abundance of conserved miRNAs from three libraries in lily**.

Supplementary Table S2 **Identification and expression analysis of novel miRNAs in lily**.

Supplementary Table S3 **Differential expression of ***B. elliptica***-responsive miRNAs in lily**.

Supplementary Table S4 **Stem-loop primers of lily**.

Supplementary Table S5 **Specific qRT-PCR primers of lily**.
