# Supplementary figures and images for: Transcriptome-Wide Analysis of Botrytis elliptica Responsive microRNAs and Their Targets in Lilium Regale Wilson by High-Throughput Sequencing and Degradome Analysis
Source: Front Plant Sci. 2017 May 18;8:753. doi: 10.3389/fpls.2017.00753 (PMC5435993; doi:10.3389/fpls.2017.00753)

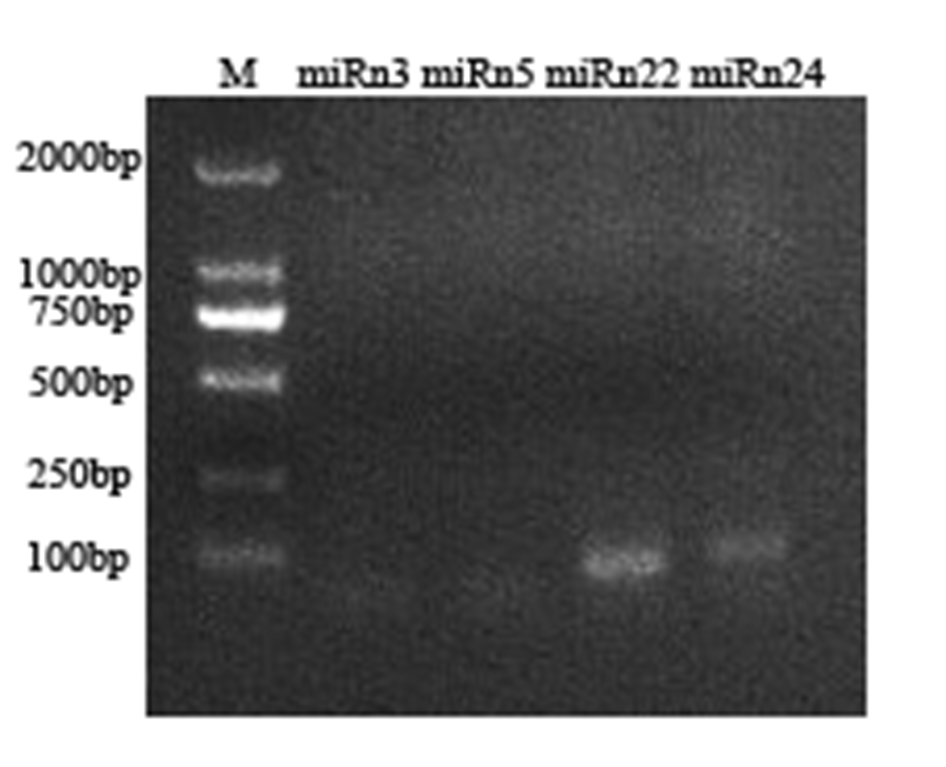

Supplement: Supplementary Figure S1 — Stem-loop RT-PCR electrophoresis of novel miRNAs. [file Image1.TIF]
